# Metagenomic and Culturomics Analysis of Microbial Communities within Surface Sediments and the Prevalence of Antibiotic Resistance Genes in a Pristine River: The Zaqu River in the Lancang River Source Region, China

**DOI:** 10.3390/microorganisms12050911

**Published:** 2024-04-30

**Authors:** Yi Yan, Jialiang Xu, Wenmin Huang, Yufeng Fan, Zhenpeng Li, Mingkai Tian, Jinsheng Ma, Xin Lu, Jian Liang

**Affiliations:** 1 School of Light Industry Science and Engineering, Beijing Technology and Business University, Beijing 100048, China; yanyi@btbu.edu.cn (Y.Y.); xujialiang@btbu.edu.cn (J.X.); amanda17806709955@hotmail.com (W.H.); 22300302071@st.btbu.edu.cn (M.T.); 2230302068@st.btbu.edu.cn (J.M.); 2 State Key Laboratory of Plateau Ecology and Agriculture, Qinghai University, Xining 810016, China; 3 National Key Laboratory of Intelligent Tracking and Forecasting for Infectious Diseases, National Institute for Communicable Disease Control and Prevention, Chinese Center for Disease Control and Prevention, Beijing 102206, China; fanyufengwork@sina.com (Y.F.); lizhenpeng@icdc.cn (Z.L.)

**Keywords:** river source basin, enrichment-based culturomics, metagenomics, antibiotic resistance genes, ARGs, Lancang River

## Abstract

Microbial communities inhabiting sedimentary environments in river source regions serve as pivotal indicators of pristine river ecosystems. While the correlation between antibiotic resistome and pathogenicity with core gut bacteria in humans is well established, there exists a significant knowledge gap concerning the interaction of antibiotic resistance genes (ARGs) and human pathogenic bacteria (HPB) with specific microbes in river source basins, often referred to as “terrestrial gut”. Understanding the microbial composition, including bacteria and resident genetic elements such as ARGs, HPB, Mobile Genetic Elements (MGEs), and Virulence Factors (VFs), within natural habitats against the backdrop of global change, is imperative. To address this gap, an enrichment-based culturomics complementary along with metagenomics was conducted in this study to characterize the microbial biobank and provide preliminary ecological insights into profiling the dissemination of ARGs in the Lancang River Source Basin. Based on our findings, in the main stream of the Lancang River Source Basin, 674 strains of bacteria, comprising 540 strains under anaerobic conditions and 124 under aerobic conditions, were successfully isolated. Among these, 98 species were identified as known species, while 4 were potential novel species. Of these 98 species, 30 were HPB relevant to human health. Additionally, *bacA* and bacitracin emerged as the most abundant ARGs and antibiotics in this river, respectively. Furthermore, the risk assessment of ARGs predominantly indicated the lowest risk rank (Rank Ⅳ) in terms of endangering human health. In summary, enrichment-based culturomics proved effective in isolating rare and unknown bacteria, particularly under anaerobic conditions. The emergence of ARGs showed limited correlation with MGEs, indicating minimal threats to human health within the main stream of the Lancang River Source Basin.

## 1. Introduction

The Lancang River, also known as the Mekong River in Southeast Asia, spans 4880 km in length, with an average annual water volume of 460 km^3^ and a flow of 15,000 m^3^s^−1^ [1]. Originating from the Three River Source Region in Qinghai Province, which also serves as the source region for the Yangtze River and Yellow River, the Lancang River holds strategic significance as an “Asian water tower”, safeguarding the ecological security of China and neighboring countries surrounded by the Qinghai-Tibet Plateau [2,3]. The Three River Source Region, a sensitive and fragile ecosystem, faces challenges such as soil erosion and continuous degradation due to variable climatic conditions and increasing anthropogenic activities [4,5]. Significant governmental efforts have been directed towards mitigating degradation and promoting sustainable regional development. River systems play vital roles in freshwater ecosystem formation and provide essential resources for both human society and ecosystems [6]. Amidst global change, including water scarcity, river pollution, and deteriorating water ecosystems, characterizing river microbial diversity within the context of changing environmental drivers becomes imperative. Microbial genetic diversity harbors substantial information supporting ecosystem functions and resilience to environmental changes [7]. Benthic bacterial communities contribute significantly to river ecosystems through biodegradation and biogeochemical cycling. However, surface-attached and bed sediment-entrained microbes often remain overlooked in broad assessments of microbial life in aquatic environments, despite their pivotal role in driving biogeochemical processes at various scales, from reach to watershed and continental [8]. Thus, understanding the composition and distribution of sedimentary bacteria communities in rivers is a critical concern in microbial ecology [9].

In the environment, the vast majority (99%) of microorganisms resist cultivation in laboratory settings [10]. Some bacteria, present in extremely low abundance, are regarded as “rare biosphere” [11], requiring cross-feeding or enrichment strategies for capture, or robust screening assays for identification. Enrichment culture, a concept dating back to mixed culture in 1895 [12], differs from culturomics, an axenic culturing approach that utilizes multiple cultivated conditions, with identification conducted via MALDI-TOF mass spectrometry and 16S rRNA sequencing followed by whole-genome sequencing of unknown species [13,14]. Enrichment culture is an effective and commonly practiced long-term strategy for increasing the population of target and rare organisms [15,16]. Both axenic and mixed cultures are effective techniques for reviving human and environmental microbial resources and are widely used for unraveling the microbial dark matter [12,17,18], particularly when uncultivated species represent a significant proportion and exhibit abundant functions [14,16]. Therefore, further cultivation and exploration to elucidate their roles in river ecosystems are of great significance [19]. However, various complex factors pose barriers to laboratory cultivation, such as pH, temperature, and pressure. Some microbes only grow under anaerobic or other extreme conditions [20], with anaerobic cultivation primarily applied in studies of the human gut microbiota [13,15,21,22], while limited research has been performed in river environments [23,24]. In our study, the anaerobic condition is highlighted for cultivating sedimentary bacteria in a pristine river.

Metagenomics has revolutionized our understanding of the relationships between the human microbiome, health, and disease, yet it has also generated a vast number of sequences that remain unassigned to recognized microorganisms [25]. While culture-independent metagenomic DNA sequence analysis offers comprehensive insights into microbial diversity on Earth, microbial isolation and cultivation remain indispensable for investigating metabolic and physiological functions, as well as ecological roles in the environment [19]. Additionally, integrating complementary methods from both culture-based and metagenomic approaches enhances our understanding of the complex ecosystem repertoire, including uncultivated species, pathogens, and the resistome, as well as host–microbiota mutualism in pristine river environments [13,26,27].

Aquatic environments host diverse microbial populations crucial for their functioning and sustainability [28]. Those polluted by residual fecal matter often exhibit increased levels of ARGs [29,30,31]. Since the conception of ARGs in 2006, they have been recognized as emerging contaminants, contributing to the contamination of aquatic environments worldwide [31,32,33]. Studies in high-elevation regions, such as the Tibetan Plateau, have indicated that direct or indirect pollution may alter the diversity and distribution of background ARGs [34], leading to the appearance of environmental resistomes. Rivers in high-elevation regions, such as the Yarlung Tsangpo River, exhibit ecological processes of ARG assembly shifting from deterministic to stochastic, with lower antibiotic levels compared to urbanized areas [35,36]. Furthermore, ARGs are often carried on Mobile Genetic Elements (MGEs) [37,38] such as plasmids, transposons, and integrons, facilitating horizontal gene transfer (HGT) between bacteria [39]. Bacteria can acquire ARGs and enhance antibiotic resistance through microbial community succession and HGT. Virulence factors (VFs) are essential components that facilitate microbial colonization and proliferation within the host, thus promoting disease progression [40]. Pathogenic bacteria universally depend on an array of VFs to initiate infections and adapt to host conditions; thereby, in-depth research on them contributes significantly to elucidating the fundamental mechanisms of bacterial pathogenesis [41]. Some pathogens in aquatic environments have become emerging pollutants containing ARGs encoding antibiotic resistance [42,43,44,45,46]. Moreover, ARGs predominantly found in pathogens pose higher risks for transmission than those residing mainly in non-pathogenic bacteria [29]. Opportunistic pathogens, organisms causing disease only in the absence of normal host resistance, are prevalent in aquatic environments [47]. For example, *Delftia* [48], *Aeromonas*, and *Pseudomonas* [49] in Yellow River sediment, and planktonic HPB like *Rheinheimera texasensis* and *Noviherbaspirillum* sp. *Root189* in the Yangtze River [27]. However, interactions between pathogens and their hosts in sediment remain understudied in these river systems.

In this study, we selected the Lancang River, a freshwater lotic ecosystem, to investigate the aquatic micro-biodiversity in sediment and the distribution of microbial communities, encompassing both anaerobic and aerobic bacteria, within sediments at an elevation ranging from approximately 3800 m to 4100 m. To our knowledge, there have not been any studies that have concurrently employed enrichment-based culturomics and shotgun metagenomic sequencing to explore the microbial diversity and dissemination of ARGs, MGEs, VFs, and pathogens in this pristine river. Our study combines the mixed culture strategy of enrichment-based culturomics, along with high-throughput sequencing to address gaps in understanding the sedimentary microbiome. The objectives of this study were as follows: (1) isolate both anaerobic and aerobic organisms to uncover scarcity and novelty in river sediment within the source region; (2) investigate the microbial composition and dissemination of antibiotics within sedimentary bacterial communities; and (3) generate preliminary profiles of ARGs, MGEs, VFs, and HPB in this pristine river. (4) The discovery of novel species helps expand the microbial biobank of culturable microorganisms and helps to explore the microbial dark matter resources in the source region.

## 2. Materials and Methods

### 2.1. Sample Collection

**Figure 1:**
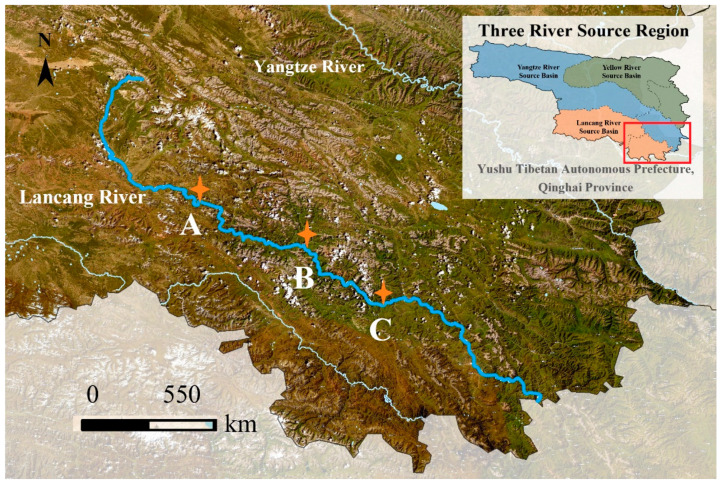
Overview and detailed sampling sites of the Lancang River Source Basin. The dots represent the surface sediment sampling sites, including site A (Upstream), site B (Midstream), and site C (Downstream).

In August 2022, three sediment samples (with three parallels per sampling site) were collected from the main stream of the Lancang River Source Basin, also known as the Zaqu River, located in Zaduo County, Yushu Tibetan Autonomous Prefecture, Qinghai Province, China (Figure 1). Three samples, named as A (*Upstream*), B (*Midstream*), and C (*Downstream*), were collected and prepared in duplicates, for metagenomic sequencing and further culturing. Surface sediments were obtained in triplets at each site using a gravity corer with an internal diameter of 50 mm, collecting sediments from depths ranging between 2 and 15 cm. Upon collection, surface sediments were transferred to sealed tubes filled with river water to maintain anaerobic conditions and were transported to the laboratory within 12 h, where they were stored at 4 °C. Additionally, another set of surface sediments was kept in the dark at 4 °C and delivered for meta-sequencing within three days. The geographic coordinates of the sediment sampling sites are as follows: *Upstream* (E95°13′, N32°53′), *Midstream* (E95°37′, N32°47′), and *Downstream* (E95°42′, N32°41′), respectively. The above sea level (ASL) of each site was measured to be 4038.7 m, 3945.0 m, and 3871.4 m, respectively.

### 2.2. Incubation of Sediment Samples

**Figure 2:**
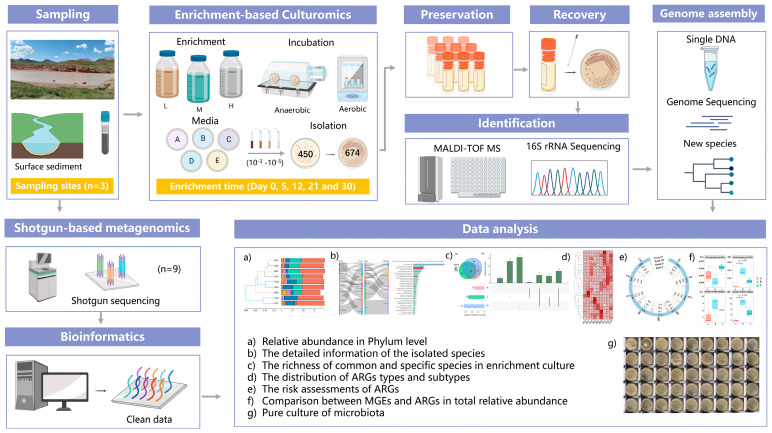
Schematic diagram of the experimental design. Three sampling sites were located in Lancang River, Qinghai Province. Triple parallels of these three surface sediment samples were collected (*n* = 9), then a mixture of the sediments was put into 3 different enrichment bottles (L, M, and H) and later incubated in both anaerobic and aerobic conditions (25 °C) for 30 days. After enrichment time (Day 0, 5, 12, 21, and 30), 674 pure cultures were generated from 450 culture media and then preserved in glycerol (30%, *v*/*v*) at −80 °C. Each strain was recovered for identification, and those 16S rRNA gene similarities <98.65% identified as potential novel species were further analyzed individually.

To comprehensively investigate the microbial community inhabiting the ecosystem, both aerobic and anaerobic conditions were set for enrichment and incubation experiments (Figure 2). A mixed culture method, enrichment culture, with certain modifications, was employed to effectively isolate potential uncultured strains from river surface sediment. This is basically an isolation technique employed to offer environmental conditions and specific nutrients, including sodium pyruvate, vitamin supplements, and trace minerals, to favor the growth of desired microbes and make them detectable [16,50]. Both eutrophic and oligotrophic media were utilized, and all 9 samples were mixed and homogenized in preparation for further enrichment culture.

To expand capacity and accurately depict the microbial community in the Lancang River Source Basin, three river sediment samples were mixed and subjected to separate anaerobic and aerobic incubation from 0 to 30 days. Enrichment culture incubation was carried out at 25 °C for 0, 5, 12, 21, and 30 days in individual 250 mL sealed glass bottles filled with 100 mL medium and 5 g of sediment sample [16]. Three enrichment media and five isolation media were selected for this study (see Table S1). For enrichment culture media, enrichment medium M, derived from marine sediment cultivation [16], contained 10 mM sodium pyruvate as the main carbon source (Solarbio, Beijing, China). Enrichment medium L was a eutrophic culture with hybrid substances [51], utilizing sodium butyrate and sodium formate as the main carbon sources (Sigma-Aldrich, Shanghai, China). Both enrichment media L and M were eutrophic media, whereas enrichment medium L underwent oligotrophic conditions with the removal of two carbon sources.

For isolation media, 5 different agar media were used to cultivate and isolate pure bacterial cultures. The selection of media was based on our previous studies and aimed to leverage each medium’s strengths for optimal isolation [52,53,54]. Media A, B, and C were oligotrophic culture media, while Media D and E were eutrophic culture media. Medium A employed the commercial medium R_2_A Agar Medium (AOBOX, Beijing, China), while medium B utilized Marine Agar 2216 (ELITE-MEDIA, Shanghai, China), originally designed for marine bacteria isolation. Medium C employed a PY agar medium for anaerobic bacteria in rice plants [55]. Medium D utilized a modified DSMZ 311c Agar medium for bacteria from freshwater sediment [56,57]. And medium E utilized a commercial PYG agar culture medium (TOPBIO, Zhaoyuan, China). 

During anaerobic incubation, three mixed culturing media were employed for enrichment. Anaerobic bacteria were incubated and isolated within an anaerobic chamber (857-OTA, Plas-Labs, Inc. ^TM^, Lansing, USA) filled with a gas mixture of N_2_-85%, H_2_-10%, and CO_2_-5%. The chamber temperature was maintained between 25 and 28 °C to optimize microbial cultivation. Meanwhile, aerobic bacteria were incubated in a laboratory shaker (Thermo Scientific™ MaxQ™ 6000, Cincinnati, OH, USA) under the same temperature conditions as anaerobic incubation.

### 2.3. Identification of Anaerobic and Aerobic Bacteria

Matrix-assisted laser desorption/ionization-Time of Flight Mass Spectrometry (MALDI-TOF MS) was applied to differentiate bacteria at the species level [58], utilizing the EXS 3000 (zybio, Chongqing, China). Fresh subcultures were grown for 24–48 h under both anaerobic and aerobic conditions to obtain sufficient single colonies for phenotypic identification. Microbes were then directly smeared onto the target plate. 

For further detection and quantification, full-length sequencing of the 16S rRNA gene was conducted using the primer pair composed of 27F (5′-AGAGTTTGATCMTGGCTCAG-3′) and 1492R (5′-GGTTACCTTGTTACGACTT-3′). Thermal cycling conditions consisted of 1 cycle at 94 °C for 3 min followed by 30 cycles at 94 °C for 30 s, 50 °C for 30 s, and 72 °C for 2 min, with a final cycle at 72 °C for 5 min. Unpurified PCR products were subjected to electrophoresis on a 2% agarose gel for detection. Subsequently, amplification products were purified using the EZ-10 Spin Column PCR Product Purification Kit (Sangon, Shanghai China) and sequenced by Shanghai Sangon Biotechnology (Shanghai, China). All PCR reactions were conducted using Premix Tap^TM^ DNA Polymerase (Takara, Beijing, China). Taxonomic identification of the 16S rRNA gene was performed using both NCBI Blast (https://blast.ncbi.nlm.nih.gov/Blast.cgi, accessed on 2 April 2023) and EzBiocloud (https://www.ezbiocloud.net/, accessed on 2 April 2023).

For single strains sharing similarities of less than 98.65% compared to known species [15], whole-genome DNA was further extracted and sequenced by Shanghai Sangon Biotechnology (Shanghai, China). Raw data were de novo and assembled by Unicycler (https://github.com/rrwick/Unicycler, accessed on 29 May 2023). Strains with Average Nucleotide Identity (ANI) < 95% and Digital DNA-DNA Hybridization (dDDH) < 70% compared to their respective type strains were considered potential novel species [59].

### 2.4. DNA Extraction, Metagenomic Sequencing, and Assembly of the Sediment Samples

Genomic DNA extraction from stream sediment samples was carried out using the ALFA-SEQ Advanced Soil DNA Kit (mCHIP, Shenzhen, China) following the manufacturer’s protocol at Guangdong Magigene Biotechnology Co., Ltd. (Guangzhou, China). The purity and integrity of DNA were accessed on 1% agarose gels, while its concentration and purity were determined by Qubit 3.0 (Thermo Fisher Scientific, Bohemia, NY, USA) and Nanodrop One (Thermo Fisher Scientific, USA).

Subsequently, sequencing libraries were constructed by ALFA-SEQ DNA Library Prep Kit (mCHIP, Shenzhen, China) according to the manufacturer’s recommendations with the addition of index codes. The library quality was evaluated via the Qubit 4.0 Fluorometer (Life Technologies, Grand Island, NY, USA) and Qsep400 High-Throughput Nucleic Acid Protein Analysis System (Houze Biological Technology Co., Hangzhou, China). Metagenomic sequencing was then performed on the Illumina HiSeq 2500 platform (Illumina Inc., San Diego, CA, USA), generating approximately 3 GB of raw reads for each DNA sample, resulting in a total data output of around 55 GB. 

Quality control of the raw reads was conducted using Trimmomatic (v 0.36). Subsequently, the clean reads were de novo assembled into contigs by MEGAHIT software (v1.2.9) [60], followed by binning of the contigs using MetaBAT. Contigs shorter than 500 bp were filtered out to ensure the qualification (≥500 bp) of predicting the open reading frames (ORFs) using MetaGeneMark (v 3.38) with default parameters. Subsequently, CD-HIT (v4.7) was employed to obtain non-redundant ORFs under the condition of 95% identity and 90% coverage. 

### 2.5. ARGs, MGEs, and VFs Annotation as Well as ARGs Risk Assessment

ARGs were annotated by pipeline software ARG-OAPs (v3.2.3), which integrates the Antibiotic-Resistance Gene Database (ARDB) and Comprehensive Antibiotic Resistance Database (CARD) [61]. Subsequently, the ARG sequences in the NCBI-NR database were integrated into the Structured Antibiotic Resistance Genes Database (SARG, https://smile.hku.hk/ARGs/Indexing/download, accessed on 12 June 2023), and the assembled sequences were identified via the HMM model. ARGs were then classified into Type, Subtype, and Gene. Potential ARGs and 16S rRNA sequences were aligned by UBLAST (with an e-value cutoff of ≤10^−7^, identity > 80%).

MGEs were calculated and annotated using an algorithm based on the Mobile Genetic Element Database (MGEdb) [62,63]. VFs were identified by blasting against the VF Database (VFDB) [41] with an identity cutoff of 70% and a coverage cutoff of 90%. To normalize the abundance values of ARGs, MGEs, and VFs (copies of ARG/MGE/VF per 16S rRNA gene) in each sample, 16S sequences were identified using the local BLAST method against the Greengenes database.

The ARG_ranker (v.3.3) computed the abundance of ARGs as the copy number of ARGs divided by the 16S rRNA gene copy number in the same metagenome, based on the Kraken 2 16S database. The health risks of ARGs were assessed using a risk framework based on three criteria: human-associated-enrichment, gene transferability, and host pathogenicity. ARG_ranker (v3.3) categorized the risk of ARGs into four ranges with a decreasing risk from Rank I to Rank Ⅳ, along with unassessed ARGs, accounting for 100% [64].

### 2.6. Composition, Correlation, and Pathogenicity Analysis

MetaPhlAn4 (v4.0.3) was employed for taxonomic profiling to study the composition of the bacterial community in each sample [65,66]. The microbial composition was characterized using MetaPhlAn4, which utilizes nucleotide BLAST (blastn) with a default e-value threshold of 1 × 10^−6^ to align reads to marker genes. Bowtie2 and SAM tools were used to determine and output the metagenomic sequence information. 

Additionally, prioritized HPB were identified according to the A-to-Z database from the National Infection Prevention and Control Manual (https://www.hartmann-science-center.com/en/hygiene-knowledge/pathogens-a-z, accessed on 10 November 2023) [67], as well as another searchable pathogen database (http://webarchive.nationalarchives.gov.uk/20121212135622/http://www.bis.gov.uk/assets/foresight/docs/infectious-diseases/t16.pdf, accessed on 15 November 2023) [68]. Pathogen Host Interaction (PHI) database (http://www.phi-base.org/, accessed on 3 December 2023) was applied to investigate the interaction between hosts and pathogens [69].

### 2.7. Statistical Analysis and Data Visualization

The Kruskal–Wallis test was adapted to compare significant differences among the three sample groups (i.e., Upstream, Midstream, and Downstream) individually. The unweighted pair-groups method with arithmetic averages (UPGMA) based on the Bray–Curtis diversity distance and the unweighted UniFrac distance was employed to determine the relative abundance of different levels. To identify the correlation and consistency of ARGs with the bacterial community (at the phylum level) and MGEs, the top 20 abundance matrix analysis of the ARG and MGE subtypes were performed by the Spearman test. 

Statistical calculations and most data visualizations were conducted using R software (v4.1.2). The Venn diagrams were generated utilizing the R package [70,71], while the Sankey diagram was visualized through the R package Sankey (v3.1.4.0). Schematic diagrams and elements in this paper were generated by BioRender (v1.0) with full publishing rights. Heatmaps were constructed using TBtools (v2.019) [72].

## 3. Results

### 3.1. River Sediments as a Microbial Biobank of Microorganisms

A total of 674 bacterial strains were successfully isolated, comprising 540 under anaerobic conditions and 124 under aerobic conditions. Importantly, ten strains from four species were identified as potential novel species; two of these strains were isolated under aerobic conditions while the remaining eight were isolated under anaerobic conditions.

**Figure 3:**
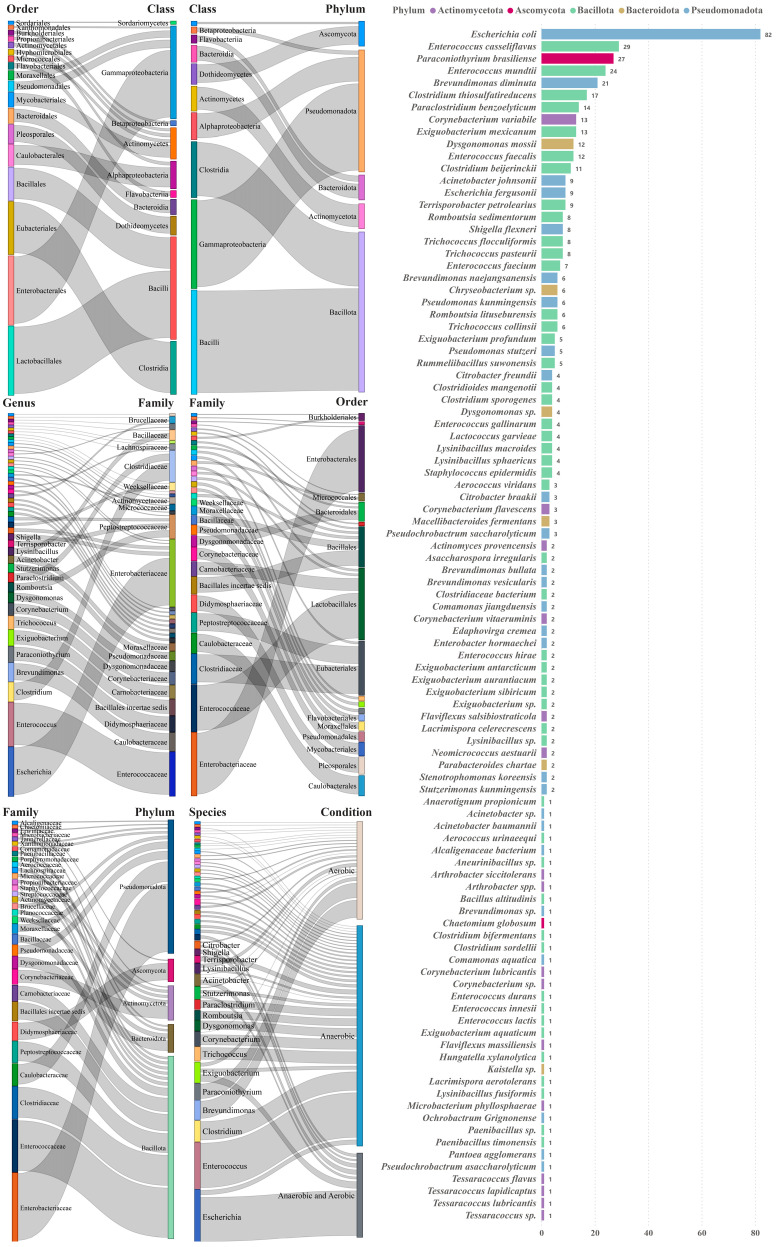
The taxonomy (phylum, class, order, family, and genus), isolation condition, and detailed information of the isolated species in sediment samples.

The richness of the isolated species was assessed at the family and genus levels, revealing a total of 5 phyla, 10 classes, 17 orders, 32 families, 48 genera, and 98 species in the pure culture isolates (Figure 3). Within the phylum of Actinomycetota, the six most frequently isolated species in the river were *Corynebacterium variabile* (*n* = 13), followed by *Corynebacterium flavescens* (*n* = 3), *Corynebacterium vitaeruminis* (*n* = 2), *Flaviflexus salsibiostraticola* (*n* = 2), *Neomicrococcus aestuarii* (*n* = 2), and *Actinomyces provencensis* (*n* = 2).

The top three species belonged to the families Enterobacteriaceae, Enterococcaceae, and Didymosphaeriaceae, as well as the genera *Escherichia*, *Enterococcus*, and *Paraconiothyrium*, respectively. In the phylum of Pseudomonadota (also part of Proteobacteria), *Escherichia coli* (*n* = 82) was the most commonly isolated species, followed by *Brevundimonas diminuta* (*n* = 21), *Acinetobacter johnsonii* (*n* = 9), *Escherichia fergusonii* (*n* = 9), and Shigella flexneri (*n* = 8). The taxonomic results from the enrichment-based culturomics indicated the successful isolation of species from four bacterial phyla, including Actinomycetota (16), Bacillota (48), Bacteroidota (6), and Pseudomonadota (26), and one fungal phylum—Ascomycota (2). Notably, the two species in the fungal phylum of Ascomycota were *Paraconiothyrium Brasiliense* and *Chaetomium globosum*, with counts of 27 and 1 strain isolated, respectively. The top five strains within the Bacillota phylum were *Enterococcus casseliflavus* (*n* = 29), *Enterococcus mundtii* (*n* = 24), *Clostridium thiosulfatireducens* (*n* = 17), *Paraclostridium benzoelyticum* (*n* = 14), and *Exiguobacterium mexicanum* (*n* = 13), respectively. *Dysgonomonas mossii* (*n* = 12), *Chryseobacterium* sp. (*n* = 6), and *Dysgonomonas* sp. (*n* = 4) were dominant in the phylum of Bacteroidota. Moreover, with a count of 82, *Escherichia coli* was the most abundant species in both anaerobic and aerobic conditions.

### 3.2. The Obtainment of Most Species Was Anaerobic and Rare

**Figure 4:**
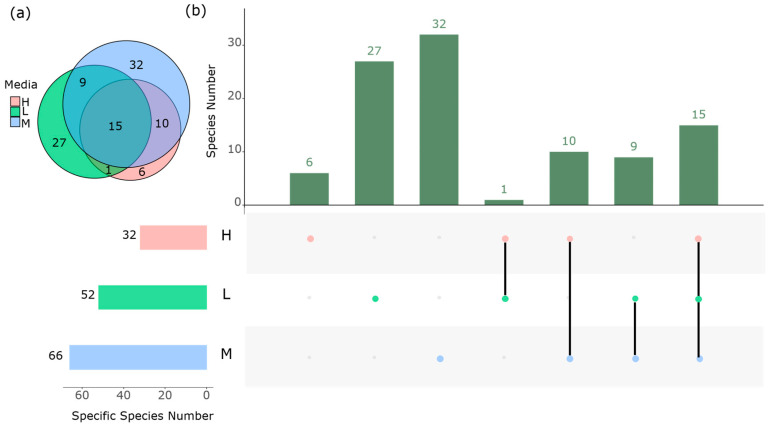
The richness of common and specific species in three different enrichment cultures. (**a**) Venn graph based on the isolated species; (**b**) the number of common and specific isolated species among three enrichment media.

Of the 674 strains that were isolated, 154 were cultured in enrichment medium H, 240 in enrichment medium L, and 251 in enrichment medium M. Additionally, 29 strains were directly cultured in the isolation media without utilizing enrichment culture media. The peak was observed on Day 5 (*n* = 238), followed by a gradual decrease over the extended culture period, with counts on Day 0 (*n* = 29), Day 12 (*n* = 166), Day 21 (*n* = 137), and Day 30 (*n* = 104) (Table S2). These findings illuminate both similarities and differences among the three enrichment culture media, as evidenced by the number of shared and unique isolated species depicted in the Venn diagram (Figure 4a). Enrichment medium M exhibited the highest diversity of species among the different media, whereas oligotrophic enrichment medium H isolated the fewest number of bacteria (Figure 4b). Moreover, the abundance of specific isolates varied across different stages of enrichment culture. 

Following a 30-day enrichment and incubation period of the surface river sediment samples, a total of 98 bacterial species were isolated. Among these, 33 species were found in aerobic environments, 58 in anaerobic environments, and 7 in both anaerobic and aerobic environments (Table S3). The seven species isolated in both anaerobic and aerobic conditions were *Acinetobacter johnsonii*, *Chryseobacterium* sp., *Citrobacter freundii*, *Escherichia coli*, *Exiguobacterium mexicanum*, *Shigella flexneri*, and *Pseudomonas stutzeri*. Interestingly, medium C accounted for 30.0% of isolated bacteria and exhibited the highest species diversity, followed by medium A (23.7%), medium B (23.0%), medium D (11.6%), and medium E (11.7%). After enrichment culture, rare species affiliated with the phyla Proteobacteria, Acidobacteria, and Gemmatimonadetes constituted a significant proportion of the total microbial community across different microcosm groups (Table S3). However, contrasting differences were observed in the phylum Cyanobacteria, particularly in the Downstream genus Pseudanabaena compared to Upstream and Midstream samples. The pure cultures isolated during different enrichment stages (Day 0–30) exhibited the following four characteristics: (1) Bacillota was the most abundant phylum after enrichment culturing, (2) the prevalence of cultivated Enterobacteriaceae family species exceeded that of other families, (3) most species were isolated under anaerobic conditions, (4) seven species were isolated under both anaerobic and aerobic conditions.

### 3.3. Human Pathogen Bacteria Existing in the River Sediment

After enrichment-based culturomics, a total of 30 HPB were identified among 98 strains (Table 1). These included various pathogens from different families and genera:

(1) Enterococcaceae family

*E. coli* possessed the largest number of bacteria isolated during the anaerobic incubation stage; *E. faecalis* and *E. faecium*, Gram-positive bacteria, were found in a significant proportion among the total strains. *Citrobacter* spp., such as *Citrobacter freundii*, was also discovered.

(2) Moraxellaceae family

*Acinetobacter* spp., including *A. johnsonii* and *A. baumannii*. *A. baumannii*, was an aerobic, Gram-negative, rod-shaped bacterium and an increasingly common cause of nosocomial infections around the world.

(3) Pseudomonadaceae family

*Pseudomonas* spp., including *P. kunmingensis* and *P. stutzeri*. *Pseudomonas* spp., is a genus of Gram-negative, rod-shaped bacteria. *P. kunmingensis* was isolated under anaerobic conditions, while *P. stutzeri* was isolated under both conditions.

(4) Corynebacteriaceae family

The results of *Corynebacterium* spp. investigated four species of pathogens, *C. variabile*, *C. flavescens*, *C. lubricantis*, and *C. vitaeruminis*. *Corynebacterium* spp. were Gram-positive, aerobic bacteria, while all of them could be isolated in anaerobic conditions in this study.

(5) Staphylococcaceae family

*Staphylococcus epidermidis* was an aerobic, spherical, Gram-positive bacterium, and was isolated in anaerobic conditions.

(6) Alcaligenaceae family

*Alcaligenes* spp. comprised Gram-negative, rod-shaped bacteria with flagella, and was isolated in aerobic conditions. Alcaligene bacteria possess natural resistance to all cephalosporins and often to aminoglycosides and aztreonam.

By metagenomic sequencing, a total amount of 532 pathogen genes and 94 types of pathogens were annotated through PHI (Table S4). Pathogen evolution often occurs when environmental organisms undergo horizontal gene transfer, equipping them with advantageous traits within their non-pathogenic habitats. A prime illustration of this phenomenon was observed during the transformation of *Vibrio cholerae* from non-pathogenic to pathogenic variants, the process of which entails the acquisition of the type IV toxin-co-regulated pilus (TCP) followed by infection with the filamentous phage CTXϕ, exploiting pilus as an entry point and introducing genes coding the production of cholera toxin. Current studies strongly indicate that specific distributed genes and gene combinations play crucial roles in determining which kinds of strains are most likely to act as pathogens [73,74,75]. The most prevalent pathogen genes included *RpoB* (belonging to *Acinetobacter baumannii*), followed by *RSc0454* and *speC* (*Ralstonia solanacearum*), as well as *CcoN2* and *rmlA* (*Pseudomonas aeruginosa*). Among the pathogens carrying the most diverse genes, the top 10 species were *Xanthomonas oryzae* (48), *Salmonella enterica* (45), *Pseudomonas aeruginosa* (40), *Xanthomonas campestris* (35), *Erwinia amylovora* (27), *Mycobacterium tuberculosis* (19), *Fusarium graminearum* (17), *Escherichia coli* (16), *Magnaporthe oryzae* (14), and *Staphylococcus aureus* (13). *Acinetobacter baumannii* and *Escherichia coli* were two of the pathogenic strains isolated in this study.

**Table 1:** The HPB existing in the isolated species, Human Pathogen Bacteria, HPB (+, pathogens; -, Opportunistic).

Num	Atmosphere	Taxonomy (Phylum-Genus)	Species	HPB	Pathogenicity and Antibiotic Resistance	Ref.
1	*Anaerobic*	*Pseudomonadota*; *Gammaproteobacteria*; *Moraxellales*; *Moraxellaceae*; *Acinetobacter*	*Acinetobacter baumannii*	-	Opportunistic nosocomial pathogen with multidrug resistance	[76,77]
2	*Anaerobic and Aerobic*		*Acinetobacter johnsonii*	+	Co-culture enhancing antibiotic resistance to Salmonella, a potentially opportunistic pathogen widely distributed in nosocomial and natural environments, and its biofilms evolve resistance to tetracycline	[78,79,80]
3	*Aerobic*	*Pseudomonadota*; *Gammaproteobacteria*; *Moraxellales*; *Moraxellaceae*; *Acinetobacter*	*Acinetobacter* sp.	+		
4	*Anaerobic*	*Bacillota*; *Bacilli*; *Lactobacillales*; *Aerococcaceae*; *Aerococcus*	*Aerococcus viridans*	+	A human and animal pathogen causing urinary tract infection, arthritis, pneumonia, meningitis, and endocarditis; Wild-type strains of *Aerococcus viridans* resistant to erythromycin, tetracycline and minocycline, chloramphenicol, and high levels of streptomycin	[81,82]
5	*Aerobic*	*Pseudomonadota*; *Betaproteobacteria*; *Burkholderiales*; *Alcaligenaceae*; *Alcaligenes*	*Alcaligenaceae* sp.	+		
6	*Aerobic*	*Pseudomonadota*; *Alphaproteobacteria*; *Caulobacterales*; *Caulobacteraceae*; *Brevundimonas*	*Brevundimonas diminuta*	-	The emerging global opportunistic pathogen	[83]
7	*Aerobic*		*Brevundimonas vesicularis*	+	Emerging global opportunistic pathogen	[83]
8	*Aerobic*	*Ascomycota*; *Sordariomycetes*; *Sordariales*; *Chaetomiaceae*; *Chaetomium*	*Chaetomium globosum*	+	Belonged to fungus and was reported as a cause of invasive pulmonary infection in a patient with Wegener’s granulomatosis.	[84]
9	*Anaerobic and Aerobic*	*Pseudomonadota*; *Gammaproteobacteria*; *Enterobacterales*; *Enterobacteriaceae*; *Citrobacter*	*Citrobacter freundii*	-	Recognized as significant pathogens in patients with underlying diseases or immunocompromised status, with multidrug-resistant strains emerging	[85]
10	*Anaerobic*	*Bacillota*; *Clostridia*; *Eubacteriales*; *Clostridiaceae*; *Clostridium*	*Clostridium sordellii*	+	Clostridial pathogen	[86]
11	*Anaerobic*		*Clostridium bifermentans*	+	A rare pathogen in humans that causes death to a young woman	[87]
12	*Anaerobic*		*Clostridium sporogenes*	+	Several findings of ARGs (VmlR2, Ard1, CplR)	[88]
13	*Anaerobic*	*Actinomycetota*; *Actinomycetes*; *Mycobacteriales*; *Corynebacteriaceae*; *Corynebacterium*	*Corynebacterium flavescens*	-	Opportunistic pathogen	
14	*Anaerobic*		*Corynebacterium lubricantis*	-	Opportunistic pathogen	
15	*Aerobic*		*Corynebacterium* sp.	+		
16	*Anaerobic*		*Corynebacterium variabile*	-	Opportunistic pathogen	
17	*Anaerobic*		*Corynebacterium vitaeruminis*	-	Resistance to vancomycin	[89]
18	*Anaerobic*	*Bacillota*; *Bacilli*; *Lactobacillales*; *Enterococcaceae*; *Enterococcus*	*Enterococcus durans*	-	Potential clinical pathogen	[90]
19	*Anaerobic*		*Enterococcus faecalis*	-	A common cause of nosocomial infections worldwide	[91]
20	*Anaerobic*		*Enterococcus faecium*	-	A hospital-adapted opportunistic pathogen	[92]
21	*Anaerobic*		*Enterococcus gallinarum*	-	Inherent resistance to vancomycin	[93]
22	*Anaerobic*		*Enterococcus mundtii*	+	A pathogen of human infectious disease	[94]
23	*Anaerobic*		*Enterococcus hirae*	+	A zoonotic pathogen rarely isolated from human infections	[95]
24	*Anaerobic and Aerobic*	*Pseudomonadota*; *Gammaproteobacteria*; *Enterobacterales*; *Enterobacteriaceae*; *Escherichia*	*Escherichia coli*	-	An opportunistic pathogen involved in various intestinal and extra-intestinal infections	[96]
25	*Aerobic*	*Pseudomonadota*; *Gammaproteobacteria*; *Enterobacterales*; *Erwiniaceae*; *Pantoea*;	*Pantoea agglomerans*	+	A plant pathogen causing human disease	[97]
26	*Aerobic*	*Ascomycota*; *Dothideomycetes*; *Pleosporales*; *Didymosphaeriaceae*; *Paraconiothyrium*	*Paracoccidioides brasiliensis*	+	Cause systemic paracoccidioidomycosis, a granulomatous disease	[98]
27	*Anaerobic*	*Pseudomonadota*; *Gammaproteobacteria*; *Pseudomonadales*; *Pseudomonadaceae*; *Stutzerimonas*	*Pseudomonas kunmingensis*	-	Opportunistic pathogen	
28	*Anaerobic and Aerobic*		*Pseudomonas stutzeri*	+	Pseudomonas bacteria are widespread pathogens that account for considerable infections	[99]
29	*Anaerobic and Aerobic*	*Pseudomonadota*; *Gammaproteobacteria*; *Enterobacterales*; *Enterobacteriaceae*; *Shigella*	*Shigella flexneri*	+	Emerging pathogenic bacteria; research on evolutionary history and antibacterial activity	[100]
30	*Anaerobic*	*Bacillota*; *Bacilli*; *Bacillales*; *Staphylococcaceae*; *Staphylococcus*	*Staphylococcus epidermidis*	+	Pathogenic bacteria, causing nosocomial infections	[101]

### 3.4. The Discovery of Potential Novel Strains Revealing Microbial Dark Matter

Ten strains belonging to four species were novel, two strains of which were isolated under aerobic conditions and eight under anaerobic conditions (Table 2). Comparative analysis in ANI and dDDH with their type strains suggested that three of these potential novel species might belong to the phylum Actinomycetota. The potential novel species identified in this study were isolated after enrichment culturing, with their type strains belonging to various families, including Microbacteriaceae, Propionibacteriaceae, Carnobacteriaceae, and Xanthomonadaceae.

### 3.5. Composition and Diversity in the Pristine River Ecosystem

**Figure 5:**
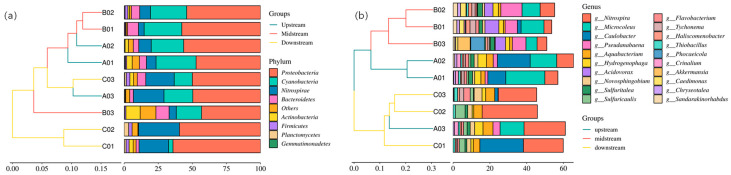
Relative abundance in phylum and genus levels. (**a**) Phylum and (**b**) Top 20 of genus level in unweighted pair-groups method with arithmetic averages (UPGMA) based on the Bray–Curtis similarity in samples from the A (Upstream), B (Midstream), and C (Downstream) areas.

After data filtering and screening, a total of 883,969,400 clean reads were generated from nine samples, with an average read length of 98,218,822 nt (Table S5). The bacterial community analysis revealed the presence of 51 phyla of bacteria and 1 phylum of archaea in all sediment samples. Regarding the bacterial community composition in the phylum level (Figure 5a), Proteobacteria (53.52%), Cyanobacteria (18.19%), and Nitrospirae (14.28%) were the most abundant phyla. Other prominent phyla included Bacteroidetes, Actinobacteria, Firmicutes, Planctomycetes, and Gemmatimonadetes.

The analysis of bacterial genera revealed a diverse microbial community, with a total abundance of 20 genera that were annotated (Figure 5b). *Microcoleus, Nitrospira,* and *Caulobacter* were identified as the three dominant genera, belonging to the phyla Cyanobacteria, Nitrospirae, and Proteobacteria, respectively. Additionally, genera within the phylum Proteobacteria, such as *Caulobacter*, *Hydrogenophaga*, *Acidovorax*, *Aquabacterium,* Sulfuritalea, and *Novosphingobium*, constituted the largest group in terms of relative abundance. Cyanobacteria was the second most abundant phylum, with genera like *Pseudanabaena* and *Tychonema* among the top 10 in relative abundance. Furthermore, a significant portion of the identified genera in the microbial community were classified as unclassified, indicating the presence of numerous unknown bacteria dominating the core microbiome.

### 3.6. Profiles of ARGs, MGEs, and VFs, and Identification of Risk Genes

**Figure 6:**
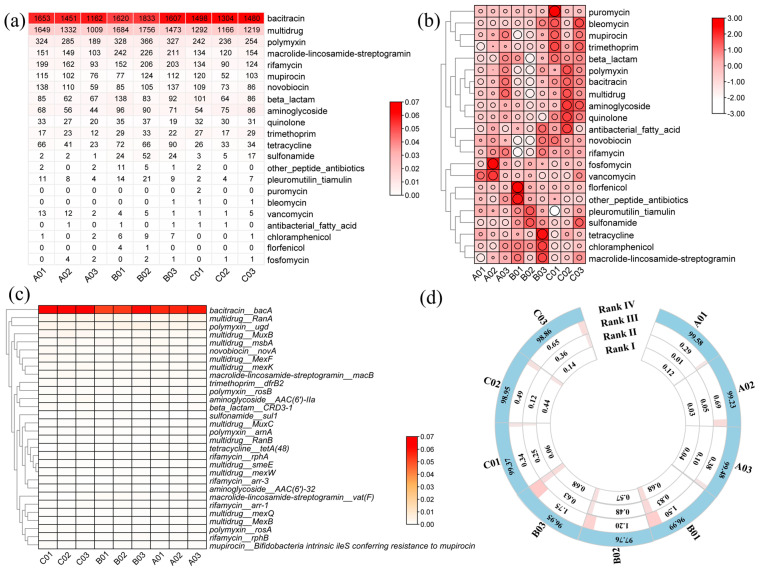
The relative abundance and total counting numbers of ARG types. (**a**) Total number of reads that mapped to ARGs and composition of ARG types in different classes. (**b**) A total of 22 types of ARG were annotated, normalized by 16S rRNA abundance (copies per 16S rRNA) and standardized by row scale. (**c**) Relative abundance of top 30 ARGs (copies per 16S rRNA) and standardized by row scale. (**d**) The risk assessments of ARGs; the percentage (%) of Rank I, Rank Ⅱ, Rank Ⅲ, and Rank Ⅳ are shown.

Overall, our analysis identified 435 ARG subtypes resistant to 22 different kinds of antibiotics across the samples. The number of ARG subtypes varied among samples, ranging from 134 in sample A03 from the Upstream to 232 in sample B01 from the Midstream. The total number of reads mapping to antibiotics was displayed without normalization by any algorithms (Figure 6a). Further analysis revealed that 12 resistance genes were present in all surface sediment samples, with a detection rate of 100% and a relative abundance exceeding 1%. These included resistant genes for multidrug (5), polymyxin (2), bacitracin (1), macrolide-lincosamide-streptogramin (MLS) (1), novobiocin (1), aminoglycoside (1), and trimethoprim (1) resistance genes (Table S6). Among these, the top three ARGs with the highest relative abundance across all samples were bacitracin (total relative abundance of 0.53 copies per 16S rRNA, accounting for 54.53%), multidrug (0.18 copies per 16S rRNA, 18.35%), and polymyxin (0.06 copies per 16S rRNA, 6.39%). In terms of diversity, ARGs resistant to beta-lactam antibiotics were the most diverse, with 141 subtypes identified. This was followed by multidrug resistance genes (80 subtypes), aminoglycoside resistance genes (46 subtypes), tetracycline resistance genes (35 subtypes), and MLS resistance genes (33 subtypes) (Figure 6b).

The abundance of the ARG subtypes varied across different antibiotics. The most abundant ARG subtype was *bacA*, which confers resistance to bacitracin, with a relative abundance of 54.29%. Other prominent ARG subtypes included *RanA* (3.86%, multidrug resistance), *ugd* (3.63%, polymyxin resistance), *MuxB* (2.31%, multidrug resistance), *msbA/mexK/MExF* (1.89%/1.70%/1.68%, multidrug resistance), *MacB* (1.68%, MLS resistance), *novA* (1.65%, novobiocin resistance), *drfB2* (1.39%, trimethoprim resistance), *AAC (6′)-IIa* (1.25%, aminoglycoside resistance), and *rosB* (1.18%, polymyxin resistance) (Figure 6c). 

Regarding MGEs, four types were identified, including plasmids, insertion sequences (ISs), transposons (Tn), and integrons (In), comprising a total of 395 subtypes (Table S7). Transposase (tnpA) and insertion sequences (IS) were the most prevalent MGE families. Additionally, 72 subtypes of ISs belonging to 20 families were identified, with IS91 being the most abundant family transposon. 

When referring to virulence factors, the top 50 relative abundance of VFs as well as their host bacteria and functions are shown in Table S8. Among these, 28 host bacteria were associated with pathogenicity-related virulence factors. Annotation results revealed eleven disease-related factor categories: adherence, iron uptake system, regulation, antiphagocytosis, secretion system, magnesium uptake system, stress protein, invasion, toxin, magnesium uptake system, serum resistance, and unclassified. The dominant VFs in this river included Type IV pili and HSI-I, both hosted by *Pseudomonas aeruginosa*. At the species level, *Pseudomonas aeruginosa* exhibited the highest abundance of VFs, followed by *Burkholderia pseudomallei* and *Aeromonas hydrophila*. Notably, *Acinetobacter baumannii*, one of the isolated pathogens in our study, was associated with the VFs of the BfmRS and AdeFGH efflux pump.

The assessment of ARGs based on the database of ARDB, CARD, and NCBI-NR [64] revealed varying levels of risk across different sites (Table S9). The majority of ARGs fell into Rank IV, representing the lowest risk category, with percentages ranging from 96.95% to 99.58% (Figure 6d). Rank I and Rank II ARGs showed similar ratios across the sites, while Rank III ARGs exhibited a slightly higher proportion in site B (Midstream) compared to sites A (Upstream) and C (Downstream). 

### 3.7. The Correlations between ARGs and MGEs

**Figure 7:**
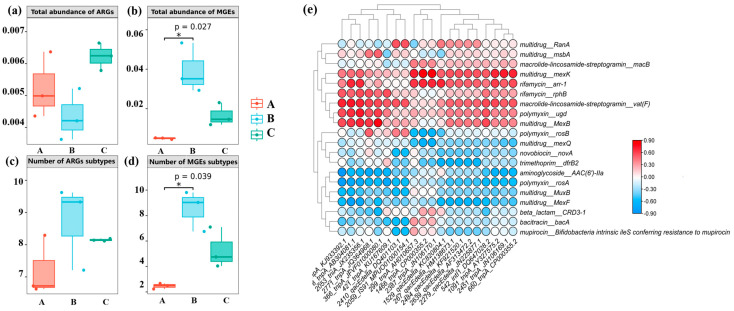
Comparison and correlation between the ARGs and MGEs (copies per 16S). Total relative abundance of (**a**) ARGs and (**b**) MGEs, the total detected number distribution differences of (**c**) ARGs and (**d**) MGEs in sites A, B, and C by the Kruskal–Wallis rank sum test, with three sampling sites as the horizontal axes and different total relative abundance and number as vertical axes. (**e**) Spearman test between the top 20 ARGs and MGEs, with MGEs as the horizontal axes and ARGs as the vertical axes.

The analysis of the total abundance and number of ARGs and MGEs provided many insights into their comparisons and correlations (Figure 7). Overall, the total abundance of MGE subtypes was higher than that of ARG subtypes. The total abundance of ARG subtypes did not show significant differences among the sites, with site C (Downstream) demonstrating the highest level (Figure 7a). Site B (Midstream) exhibited the highest total abundance of MGE subtypes, significantly higher than the other sites, while site A (Upstream) showed the lowest total abundance of MGE subtypes but was the most representative of the three sites (Figure 7b). Regarding the number of subtypes, the overall number of ARG subtypes ranged between 6 and 10 in each sample (Figure 7c), while the number of MGE subtypes ranged between 2 and 10. Notably, the number of MGE subtypes exhibited significant differences among the sites, with site B possessing the highest number (Figure 7d). Correlation analysis suggested subtle correlations between the total abundance of ARG and MGE subtypes, as well as a relatively similar connection between the number of ARG and MGE subtypes. 

The correlation analysis of the top 20 abundance matrices of MGEs and ARGs at the subtype level was performed by the Spearman test (Figure 7e, Table S10). Different ARG subtypes (*AAC (6′)-IIa*, *MuxB*, *MexF*, and *rosA*) shared negative correlations with different types of MGE subtypes (*TnpA*, *qacEdelta*, *tniA*, and *intl1*) while the remaining MGEs showed little significance and correlation with ARGs. However, the Spearman test results indicated that only a small percentage (5.75%) of the correlation tests showed significant correlations (*p*-value < 0.05). Most of the ARG and MGE subtypes exhibited negative correlations, indicating potential interactions between ARGs and MGEs.

## 4. Discussion

This study focused on the Zaqu River, namely the main stream of the Lancang River Source Region, which plays a crucial role in connecting continents and oceans, akin to the concept of a “terrestrial gut” [27]. This aquatic ecosystem serves as a reservoir for antibiotic resistance and hosts diverse environmental biomes [30]. 

One of the key highlights of this research was the effectiveness of the enrichment-based culturomics strategy in expanding the diversity of cultivable microbial communities in river surface sediment. This strategy, validated by previous studies, involves two stages: the Enrichment Stage and the Isolation Stage. During the Enrichment Stage, bacteria in the natural environment engage in competition for nutrients and minerals, with oligotrophic media proving favorable for their growth [50]. In the subsequent Isolation Stage, providing precise nutrients promotes better growth, with PYG agar being particularly effective. Traditional culturing methods lacking enrichment stages yielded less success in isolating certain bacteria, underscoring the significance of enrichment-based approaches. Our findings, in conjunction with insights from previous studies [16,50,106,107,108], emphasized the effectiveness of the mixed culture strategy of enrichment-based culturomics for exploring uncharted environments and isolating novel and rare species. This approach facilitates the creation of simplified microbial communities amenable to rational and robust study [12].

However, there have also been some limitations in bridging real existing bacteria with laboratory-cultivated ones. Dominant bacteria such as *Escherichia coli* [109,110] in the phylum of Proteobacteria tend to monopolize ecological niches, while other species in the phylum of Gemmatimonadetes [111] struggle due to their disadvantaged position, leading to their failure in isolation. Optimization of our strategy should be further warranted to enhance our understanding of the microbial biobank.

The research unveiled four potential novel species, underscoring the efficacy of this approach in unearthing new microbial taxa. Compared with their type strains, four potential novel species with 16S rRNA gene sequence shared a similarity of less than 98.65%. The type strains of the four potential novel species in this study were *Ancrocorticia populi sk1b4* (T) [104], *Jeotgalibaca porci CCUG 69148* (T) [105], *Brooklawnia cerclage BL-34* (T) [103], and *Leucobacter humi Re6* (T) [102]. Further investigations will delve into their morphological, phenotypic, and chemotaxonomic characteristics.

By mapping metagenomic shotgun sequencing data, our study revealed Proteobacteria as predominant in this pristine river, corresponding with a former study on river ecosystems [112]. Additionally, the analysis of ARGs indicated relatively low levels in the pristine river compared to areas with higher anthropogenic activities [6]. For instance, *bacA* emerged as one of the most dominant ARGs in river water and sediment samples [113]. Similarly, *bacA* was also the most detected ARG gene in the Yarlung Tsangpo River [31] and Hanjiang River Basin [114]. Its dominance has been reported in various environmental contexts, such as mariculture coastline [115], landfill leachate [116], and large cascade reservoirs [112], owing to its intrinsic presence and homologs across diverse genera [31].

Apart from *bacA*, the relative abundance of ARGs among each group showed minimal differences. For instance, the *macB* gene was reported to be associated with expelling antibiotics and exporting VFs from Gram-negative bacteria in a previous study [117]. Multidrug efflux pumps and the isolated bacteria detected in river water, such as *Pseudomonas aeruginosa* and *Escherichia coli*, were also observed. These isolates belonged to the families of *Pseudomonadaceae* and *Enterobacteriaceae* [118,119], respectively. Metagenomics results indicated *E.coli* as a major pathogen, whereas *Pseudomonas aeruginosa* was not observed (Table S5). The detection of the *macB* gene suggested that the microbes in river sediment have the potential to inhibit the multidrug efflux pump, affecting diverse cellular processes to mitigate antimicrobial resistance [117,118]. These results also implied that the ARG profile in this pristine river compared to those in urbanized areas remained at a relatively low level, suggesting limited anthropogenic activities [120].

The health risk of ARGs was evaluated based on previous research frameworks [64], assessing the risk of antibiotic resistance in human accessibility, mobility, pathogenicity, and clinical availability. Compared to site C and site A, the Midstream exhibited higher ARG levels, with site B situated near the downtown area of Zaduo County. The health risk assessment of annotated ARGs highlighted low risks to human health at the sampled sites, reflecting the preservation of a pristine environment. Moreover, it was suspected that in river systems, high ARG risks may be associated with MGEs or supercarriers of human pathogen bacteria [27]. The highest risk rank among these samples was Rank Ⅳ, indicating the least likelihood of endangering human health. Given that the sampling sites are located within the Three River Source Region ecosystem, which holds socioeconomic and regional significance [2], the well-preserved surface sediment environment in this pristine river, with limited harmful ARG dissemination, bodes well for environmental and One Health frameworks [121]. 

MGEs such as ISs were identified, indicating potential mechanisms for horizontal gene transfer and virulence evolution. ISs, reliant on transposase genes (*Tnp*), facilitate horizontal gene transfer, enabling bacteria to acquire multiple resistances [122]. IS91, the prototype element of a family of bacterial insertion sequences, transposes via a rolling-circle mechanism [123]. Family elements are often found adjacent to pathogenicity- and virulence-related genes, playing crucial roles in the dissemination and evolution of virulence [124]. Unlike previous studies annotating virulence genes in the Yarlung Tsangpo River [6], our study emphasized pathogenicity- and function-related factors of VFs. For instance, with *Pseudomonas aeruginosa* as their host bacteria, Type IV pili (i.e., adherence) may attach to host cells, induce twitching motility, or assist in biofilm formation, while HSI-I (i.e., secretion system) may contribute to chronic *P. aeruginosa* infections. Moreover, VFs of *Acinetobacter baumannii*, such as the AdeFGH efflux pump (i.e., unclassified category), may play roles in synthesizing and transporting autoinducer molecules during biofilm formation, and BfmRS (i.e., regulation) may control biofilm formation, cellular morphology, adhesion, serum resistance, and antibiotic susceptibility.

Overall, this study provides valuable insights into sedimentary bacterial diversity and highlights the need for further research, particularly in understanding planktonic bacteria and their interactions with ARGs and HPB. Future studies will employ metagenomic assembly techniques to explore ARG-carrying contigs and metagenome-assembled genomes (MAGs), advancing our understanding of antibiotic resistance in natural environments. Moreover, sampling sites of spatiotemporal and geographical variations along the river could provide comprehensive insights into microbial communities and ecological processes.

## 5. Conclusions

In this study, the research focused on a previously unstudied pristine river in the Lancang River region, employing a preliminary mixed culture strategy called Enrichment-based culturomics. The profiles and diversity between sediment bacterial communities, ARGs, MGEs, and VFs were comprehensively characterized by metagenomic sequencing. The result suggested that novel and rare species were isolated, with HPB existing in the river. Moreover, disseminated ARGs posed low risks to humans and the environment, indicating minimal influence from anthropogenic activities on the pristine river. Overall, this study contributes to a better understanding of microbial resources, ARG dissemination, and HPB in the source region of a pristine river, also underscoring the importance of environmental preservation within the One Health framework.

## Figures and Tables

**Table 2:** The protologue of 4 novel taxa in the biobank (rank indicates the type of novel, nov.: novel).

Name	Type Strain	Rank	Num	Isolation Condition	ANI (%)	dDDH (%)	16S rRNA Gene Sequence Similarity (%)	Ref.
XY-01	*Leucobacter humi Re6(T)*	Species nov.	2	Aerobic	74.38	14.20	98.01	[102]
XY-02	*Brooklawnia cerclage strain BL-34(T)*	Species nov.	6	Anaerobic	75.77	17.30	97.48	[103]
XY-03	*Ancrocorticia populi strain sk1b4(T)*	Species nov.	1	Anaerobic	77.12	21.30	97.27	[104]
XY-04	*Jeotgalibaca porci strain CCUG 69148(T)*	Species nov.	1	Anaerobic	77.28	23.50	97.79	[105]

## Data Availability

The raw sequence data of metagenomes analyzed in this paper have been deposited in the Genome Sequence Archive (GSA) in the China National Center for Bioinformation, under GSA number: CRA014594.

## Supplementary Materials

The following supporting information can be downloaded at: https://www.mdpi.com/article/10.3390/microorganisms12050911/s1, Table S1: The ingredients of enrichment and isolation culture. Table S2: The microbial biobank of enrichment-based culturomics. Table S3: The taxonomy, richness, and abundance of the isolated species. Table S4: the pathogen–host interaction and functions. Table S5: Metagenomic sequencing information. Table S6: Abundance of ARG subtype and its type. Table S7: Relative abundance of MGE subtype. Table S8: Top 50 of the relative abundances of virulence factors. Table S9: The risk percentage of ARGs. Table S10: The correlation analysis of the top 20 abundance matrices of MGEs and ARGs.

## Abbreviations

ARDB	Antibiotic-Resistance Gene Database
ARGs	Antibiotic resistance genes
CARD	Comprehensive Antibiotic Resistance Database
HGT	Horizontal gene transfer
HPB	Human pathogen bacteria
MAG	Metagenome-assembled genome
MGE	Mobile Genetic Elements
MGEdb	Mobile Genetic Element Database
SARG	Structured Antibiotic Resistance Genes Database
VF	Virulence factors
VFDB	VF Database
